# Philadelphia Hospital

**Published:** 1843-01-21

**Authors:** J. P. Tabb

**Affiliations:** Resident Surgeon


### PHILADELPHIA HOSPITAL.

### PROFESSOR PANCOAST'S CLINIC.

January 14, 1843.

[Reported by J. P. Tabb, Resident Surgeon.]

Dr. Pancoast began the morning's clinic with the exhibition of a case illustrative of the effects of bad surgery. The patient, in a row at the West, had his left ulna broken at its upper third, and the head of the radius dislocated and thrown forwards upon the outer condyle of the os humeri, shortening the fore-arm, and in consequence producing an angular deformity of the ulna at the place of fracture. In this condition the limb was put up in splints; no reduction of the radius having been effected. From the want of coaptation of the ends of the ulna, union has taken place merely by fibro-ligamentous matter, and a false joint is formed. From this cause and the impaired motion of the elbow joint, the limb has become nearly useless. The patient states that an operation has been performed without success for the pseudo-arthrosis. An opportunity being now afforded by a gentleman from the West, to obtain a history of the case, any proceedings in it will be deferred to a subsequent clinic.

Case II.—*Injury of the head.* The subject of this injury while in a fit of intoxication fell from a wagon on the back of his head, was taken up stunned, and the next day brought to the wards of the hospital for a contusion of the scalp. On entry he complained of no pain, seemed in good spirits and gave a jocular account of the mishap. On the third day after the injury, Jan. 1st, complaining of dull pain in the head, and appearing stupid, while his pulse was rather feeble, blisters were applied to the back of the neck and he was freely purged. This treatment improved him, and for several days he continued to get better, but on the morning of January 5th he became delirious and was disposed to roll out of his bed. The delirium after a few hours was followed by coma to such an extent that he could not be aroused; rigidity of the left arm was observed, paralysis of the upper lid of the same side, and dilatation of the right pupil. The muscles of the left side of the tongue were palsied, so that the point of this organ was drawn to the right side. There were no convulsions or spasms; the face was pallid, and the pulse regular, slow and feeble. Four cut cups were ordered to the back of the neck, a stimulating injection of oil of turpentine and tincture of assafœtida, stimulating pediluvia and sinapisms to the lower extremities. Under these applications he revived; was delirious in the early part of the next day, and in the evening he again became comatose; stimulating injections and pediluvia were again resorted to with success, the rigidity of the arm diminished in intensity and became intermittent, no paralysis existing except that of the right eye lid, and the tongue. The right pupil was still more dilated than natural, but the functions of the retina seemed unimpaired. His intellect was dull, but he was now capable of giving correct answers when fully aroused. By the occasional repetition of these measures, the administration of mild nutriment, and a second blister to the nape of the neck, the patient has continued to improve slowly but steadily.

The patient is not yet, however, out of danger, from results that often follow severe blows upon the cranium, and sometimes at a later period than this.

This case, the lecturer observed, was one well deserving of attention, as a representative of a class of affections which come frequently under the charge of the practitioner, and would well convey to the minds of the gentlemen present, some idea of the obscurities and difficulties often attending these interesting affections.

The governing symptoms of this case have been those of concussion, accompanied by a severe contusion of a portion of the substance of the brain. The evidence of the latter being found in the rigidity of the muscles of the arm, in the local palsy, and in the absence of the more fixed signs of concussion; but there were some symptoms of meningeal inflammation in the delirium, and of compression in the coma and palsy that followed. But the two latter were transient, and followed (not in the usual order in such cases,) by the signs of concussion, a state in which the face is pale, the pulse feeble, the breathing slow and weak, and in which the patient, when thoroughly aroused, gave correct answers.

The lecturer took occasion to observe, that the symptoms were often thus involved, and great caution was requisite in reference to the prognosis. He considered concussion a sort of instantaneous compression of the cerebral mass, and referred to the experiment of Gama, in which a transparent glass matrass, containing some curled hair, was filled with a solution of icthyocolla, which, when cooled, was about the consistence of the brain. When the glass was struck, the contained mass instantaneously left that side, and was compressed in its diameter; the effect of the blow on the glass transmitted to the opposite wall was such as to drive back and compress the mass in the opposite direction, and the force of the shock was finally spent as shown by the vibration of the hair in the mass of the contained substance. All this takes place with great rapidity, so as to be nearly instantaneous. When violence, in like manner, is applied upon the surface of the skull, in addition to the shock of the brain, which destroys for the time its functions, you may have without fracture of the bones the small vessels which connect the dura mater and the bone broken, or the middle meningeal artery torn off, if the blow be made over the temples, or one of the large venous sinuses ruptured, or a laceration of the substance of the brain. From any of these causes blood may accumulate, which, after a time, gives rise to symptoms of compression. This will take place in a few hours when the large artery is torn; or more slowly and to much less extent if one of the venous sinuses is ruptured, as the venous blood flows less freely, is sooner arrested, and is less disposed to coagulate. Compression may come on more slowly after phrenitis, from the secretion of serum or pus, or even at a later period than this from the occurrence of the injury, when the contusion of the brain has been followed by abscess. In the case before us, from the order of the symptoms, it would appear that beside the concussion and contusion there may have been some limited effusion, perhaps from the vessels of the dura mater, or it may be, as the shock was received over the occipital protuberance, from the laceration of one of the venous sinuses. But the effusion, if it exist, must be limited, and will probably be taken away by the absorbents. Though the *exact* diagnosis in this case is difficult, the practice to be pursued is very obvious. The simple measures resorted to, such as common sense would dictate, give promise of complete success. The palsy is disappearing, and the intellectual faculties, though slow in their operation, seem sound. The lecturer took occasion to observe, that though where there is flushed face, fever and delirium, following concussion, blood must be drawn boldly and frequently; yet indiscriminate bleeding in cases where the power of the brain remained more or less enfeebled after concussion, was often productive of great injury. He had several times seen instances where, after injuries of the cranium, bleeding combined with the tartar emetic practice of Dessault, had been so boldly resorted to, to *prevent* inflammation, that the brain was robbed of so much of its blood that it lost the power to recover from its shock—the *brain*, on post-mortem examination, looking blanched, like that of a calf slaughtered at the shambles. Even where there is reaction, not disposed to run high, assafœtida, or even laudanum, in the form of injection, will occasionally be useful in controlling the nervous disturbances resulting from the concussion.

The lecturer referred to a case illustrative of this treatment strictly in reference to the symptoms, which he had attended in conjunction with Dr. I. Parrish of this city. It was that of a lad who had fallen from the fourth story of a store through the hatchway into the cellar, and struck with his head obliquely upon a pile of bricks. The force of the fall was somewhat diminished by coming in contact with the margin of the hole in the second story, which broke the humerus. The patient lay for many days in a state of exhaustion, with occasional feeble efforts at reaction. But a few ounces of blood was taken during the treatment, which was analogous to that above described. The recovery in that case was perfect.

In the case before us, there was no depression of the bone—no apparent fracture—no pulpy tumour of the scalp, or separation of the periosteum at the place of the wound—no paralysis of the muscles of the palate, or of the sphincter of the rectum, or detrusor muscles of the bladder, causing stertorous breathing, involuntary discharge of fæces, or retention of urine, indicative of extensive effusion within the skull; or in other words, no possible indication for the use of the trephine, an instrument never to be employed save where it is very clearly called for. He exhibited those automatic movements of the hand to the head, once believed as serving to show the seat of the compression. But this is a symptom common to various cerebral affections, and now considered of little or no importance in the diagnosis.

Case III.—A female in the wards of Prof. Dunglison, labouring under strangulated crural hernia, was brought forward at the close of the morning's clinic, and the operation for the relief of the intestine performed before the class. The lecturer, while preparations were being made, observed that this case had but just come under his notice, the patient having entered the hospital but a short time previous. The account which she gives of herself, is as follows:

From an early period of her life, she has suffered with crural hernia, and has never worn a truss. She has been subject to frequent hernial protrusions, but the viscera were usually readily returned. Latterly she has suffered more on these occasions; violent pains and vomiting accompanying the descent of the intestine, which could only be returned by the aid of a physician. Each successive protrusion had for some time past, been more distressing, and more difficult of relief; and some of the contents of the sac had become adherent, or could not be returned. This is probably omentum. The one under which she is now suffering, occurred three days ago. It has been attended with severe and frequent vomiting, obstinate constipation, and violent pain at the seat of the tumour, with feelings of great languour and exhaustion. Finding herself gradually getting worse, and all attempts by taxis on the part of her physician failing, she has been brought into the hospital.—During the short period that has elapsed since her arrival here, the vomiting has ceased But her condition is far from being improved; the tumor is unreduced—the pulse is rapid and feeble; there is much tympanitic distension of the bowels, and soreness of the abdomen under pressure, especially about the umbilicus, indicative of commencing general peritonitis, and rendering the result in this case doubtful. The operation is therefore the only chance for her relief, and no time is to be lost in its performance. There is no danger to be dreaded from the nature of the operation when properly performed; but there is great danger already upon us, from the length of time which has already elapsed. The size and shape of the tumour is somewhat peculiar; but such as are occasionally seen in old cases of crural hernia, where the pouch of peritoniun which forms the sac and remains down, is gradually expanded by the successive protrusions of the hernia, and extends between the superficial and deep fasciæ of the thigh, in the direction where there is least resistance—towards the anterior and superior spinous process of the ilium. It now forms a tumour, such as can be grasped by the hand, about five inches in length underneath, but slightly overlapping Poupart's ligament. From the rounded shape of the inner end of the tumour, Dr. P. was induced to open the skin by a semilunar incision of two inches and a half, concave towards the vulva. This flap of skin, after the other coverings were cautiously divided, was drawn towards the other side, and gave a distinct view of the contained parts. The sac was principally filled with a fluid of a light chocolate color, and was divided as it were, into two pouches, by a middle vertical septum. A small knuckle of intestine of a mahogany hue, lay at the back surface of the crural ring. Embracing it completely on its inner and anterior surface, was a process of omentum, completely adherent, terminating in round hardened masses, which overhung the strangulated intestine. The lining membrane of the sac, and the surface of the omental tumours, were mottled with inflammation. The omentum had evidently been for a long time irreducible; and from its thickening round the margin of the ring, had evidently been the cause of the greater suffering which the patient experienced when the intestine slipt out below it, as well as that of the present strangulation. It had also narrowed the ring so as to prevent such a descent of intestine as to fill the whole sac, causing it to become strangulated when a small knuckle had passed, as in the ordinary forms of crural hernia. From the adhesion of the omentum to the sac, it was found impossible to reach the place of stricture, without cutting through the omentum. Dr. Pancoast decided to attempt its division on the outer side of the sac, which was accomplished with little difficulty. The upper margin of the sac was drawn downwards, and the cellular tissue above it separated with the point and handle of the scalpel, till Gimbernat's ligament was fairly brought into view. This was punctured in the middle, and divided from above downwards, with a probe pointed bistoury. Two firm bands below this, believed to be Hey's ligament, and the thickened sheath of vessels, were successively raised up on the nail of the forefinger of the left hand, and cautiously cut in like manner. Perfect relief to the strictured parts were thus afforded, without the lining membrane of the sac at the place of the stricture or the omentum being injured with the knife. The intestine was slightly drawn forwards and examined. There was no cadaveric odour emitted—no phlyctenæ, grey spots or removal of its peritoneal coating. It presented a white line at the place of stricture, but without softening. Its dark colour appeared to be owing to effused blood under the serous coat. Though the colour did not appear to improve but little after the removal of the stricture, Dr. P. concluded, in the absence of the signs of gangrene, to return the intestine, which was done without difficulty. The adherent portion of omentum, was left in the sac, and the wound closed with the interrupted suture, and dressed with a compress and spica bandage. The patient suffered little pain during the operation, and expressed herself much relieved at the close. She was directed to be kept quiet, with her thighs flexed, and take a desert spoonful of castor oil every hour till her bowels were freely moved: an enema of aloes and asafœtida to be given in the course of a couple of hours.

☞ We have been compelled, through want of space, to defer a portion of this clinical lecture, comprising several operations, to the next number.

